# Composite root-soil mechanics of native vegetation in Central-Western Inner Mongolia

**DOI:** 10.7717/peerj.21211

**Published:** 2026-05-28

**Authors:** Zhi Hui Ning, Ge Ri Le, Lu Ya Hui

**Affiliations:** College of Desert Control Science and Engineering, Inner Mongolia Agricultural University, Hohhot, China

**Keywords:** Root-soil composite, Shear strength, Cohesion, Internal friction angle, Soil moisture content

## Abstract

This study aims to enhance the biomechanical database concerning nitrogen fixation and plant erosion resistance in arid and semi-arid regions, providing a scientific foundation for the selection of native shrub and herb species and the optimization of mixed planting patterns for desertification control and reclamation of the Haidaigou open-pit coal mine dump in central-western Inner Mongolia. Five typical native species were examined: the shrubs *Caragana korshinskii* and *Hippophae rhamnoides*, the semi-shrub *Hedysarum fruticosum*, and the herbs *Medicago sativa* and *Astragalus adsurgens*. Experiments utilized loess soil from the dump site, characterized by a natural moisture content of 7.5% and a dry density of 1.56 g/cm^3^, with local field soil serving as a control. A ZJ-type strain-controlled direct shear apparatus was employed to test root–soil composites containing fine roots (1–1.5 mm in diameter) across five moisture content levels (3.5%, 7.5%, 11.5%, 15.5%, and 19.5%), all below the saturation point of 21.8%. Key mechanical parameters, including shear strength, cohesion, and internal friction angle, were measured under vertical loads of 12.5 kPa (simulating an 80 cm soil layer) and 25 kPa (simulating 150 cm). Results indicated that, at natural moisture content, the increase in shear strength of root–soil composites was greatest for *Caragana korshinskii* (60%), followed by *Astragalus adsurgens* (49%), and lowest for *Hedysarum fruticosum* (29%). Cohesion exhibited the highest increase in *Astragalus adsurgens* (46%) and the lowest in *Hippophae rhamnoides* (28%). As soil moisture content increased, both shear strength and cohesion initially rose before declining, with species-specific optimal moisture levels identified as follows: 7.5% for *Caragana korshinskii*, 11.5% for *Hippophae rhamnoides*, 7.5–11.5% for *Medicago sativa*, 11.5% for *Hedysarum fruticosum*, and 7.5–11.5% for *Astragalus adsurgens*. The internal friction angle of root–soil composites decreased slightly (–1% to –25%) compared to the control, confirming that cohesion is the primary contributor to the enhancement of shear strength. Under a 12.5 kPa load, root-induced soil consolidation was pronounced; however, at 25 kPa, this effect diminished, likely due to sparse root distribution in deeper layers. Furthermore, mixed-species plantings enhanced surface soil mechanical stability more effectively than single-species stands. These findings elucidate the biomechanical behavior and moisture response of root–soil complexes in native plants of arid and semi-arid regions, providing scientific and technical support for optimizing mixed “shrub– semi-shrub –herb” configurations in desertification control and mine reclamation.

## Introduction

Arid and semi-arid regions are critical areas for global desertification control. In northern China, the central and western parts of Inner Mongolia serve as a vital component of the ecological security barrier. These areas, characterized by loess hilly landforms and disturbed sites such as coal mine waste dumps, are particularly susceptible to severe soil erosion and slope instability. Biological measures, specifically vegetation soil stabilization, have emerged as the primary technology for soil and water conservation and desertification mitigation in this region. This approach is favored for its dual roles in ecological restoration and slope stabilization (Zhang et al., 2025). The fundamental mechanism of plant soil fixation involves the formation of a root-soil complex, which enhances erosion resistance and slope stability by influencing mechanical properties such as soil shear strength, cohesion, and internal friction angle. This process is influenced by the interplay of plant species, root morphology, and soil moisture conditions (Su et al., 2021; Jiang et al., 2015). Understanding the interactions among these factors is essential for optimizing vegetation protection technology. The mechanical properties of root-soil complexes have become a research hotspot in the field of vegetation soil fixation. Scholars at home and abroad have carried out a series of studies on the influence of soil moisture and plant types on the stability of root-soil complexes. Studies have confirmed that soil moisture can indirectly regulate the stability of the root-soil complex by altering the bonding state of soil particles and the flexibility of root systems (Wang et al., 2025; Zhou et al., 2024); The characteristics of root system quantity (root length density, root diameter distribution) are the core factors determining the differences in the shear resistance capacity of soil (Gerile et al., 2018); The flexural mechanical properties of native shrubs in the central and western parts of Inner Mongolia are significantly positively correlated with their soil fixation efficiency, providing a fundamental basis for the screening and application of native plants (Cui et al., 2021).

Despite the accumulation of relevant research, significant gaps remain in the study of artificially disturbed sites in arid and semi-arid regions. First, existing studies predominantly focus on individual plant species or specific water conditions (Cui et al., 2021; Kang et al., 2021), which limits systematic comparisons among native plants with varying life forms, such as shrubs, semi-shrubs, and herbaceous plants. This lack of comparative analysis hinders the scientific configuration of mixed vegetation. Second, there is inadequate investigation into the water response mechanisms of the root-soil complex in reclaimed sites, such as coal mine waste dumps, including yellow soil reclamation areas. Although prior research has established that the root systems of herbaceous plants can enhance the anti-sliding capacity of shallow soil through the “network effect” (Lv et al., 2024), studies comparing the mechanical properties and water adaptability of root-soil complexes among typical native herbaceous plants, such as *Medicago sativa* and *Astragalus adsurgens* in central and western Inner Mongolia, and local shrubs and semi-shrubs remain scarce. Consequently, a precise biomechanical basis for vegetation configuration in reclamation areas cannot be established (Yang et al., 2022; Meng, Zhao & Yang, 2020).

The waste dump of the Heidai Gou open-pit coal mine exemplifies the artificially disturbed landforms characteristic of this region. The reclaimed soil, known as yellow sponge soil, exhibits low fertility and a loose structure. The vegetation used for reclamation must fulfill the dual requirements of native adaptability and mechanical efficiency for soil fixation (Chen et al., 2025). Current research has primarily focused on the root-soil interface characteristics of individual native plants and specific soil types (Zhang, 2019; Zhu, 2016), neglecting the comparative analysis of multiple life forms and the effects of varying water gradients. While some studies have highlighted the significance of root system soil fixation for slope stability, they have not elucidated the mechanical advantages of different life forms under identical reclamation site conditions (He, 2024; Lyu et al., 2025). Notably, the abrupt changes in soil moisture content resulting from front-type rainfall are a primary cause of landslides in waste dumps (Jiang et al., 2025). However, the response patterns of root-soil complexes from various life forms of native plants to fluctuations in moisture content remain poorly understood, presenting a critical challenge to the precise configuration of reclamation vegetation.

This study addresses identified research gaps by proposing a core hypothesis: significant differences exist in the mechanical properties of root-soil complexes among native plants of varying life forms, specifically shrubs, semi-shrubs, and herbaceous plants. Furthermore, these differences exhibit distinct response patterns along gradients of soil moisture content. Notably, mixed plant combinations may enhance the stability of root-soil complexes through complementary root morphology. To investigate this hypothesis, five typical native plants from the central and western regions of Inner Mongolia were selected as research subjects: the shrubs *Caragana korshinskii* and *Hippophae rhamnoides*, the semi-shrub *Hedysarum fruticosum*, and the herbaceous plants *Medicago sativa* and *Astragalus adsurgens*. The study utilized reclaimed yellow cotton soil from the Heidai Gou open-pit coal mine waste dump as the substrate. The mechanical properties of the root-soil complex, including shear strength, cohesion, and internal friction angle, were analyzed under varying soil moisture content gradients using an indoor direct shear test system, with plain soil serving as the control. The research aims to: (1) enhance the biomechanical database of root-soil complexes associated with native plants in reclaimed sites located in arid and semi-arid regions; (2) elucidate the water response mechanisms of root-soil complexes in native plants exhibiting various life forms; and (3) establish a scientific foundation for the mixed vegetation configurations necessary for desertification prevention and control in this area, as well as for the reclamation of coal mine waste dumps. The findings of this study hold substantial theoretical significance for advancing the understanding of slope stability mechanisms in vegetation construction and for clarifying the coupling relationship between soil moisture and the mechanical processes involved in root soil fixation.

## Materials and Methods

### Study site

**Figure 1:**
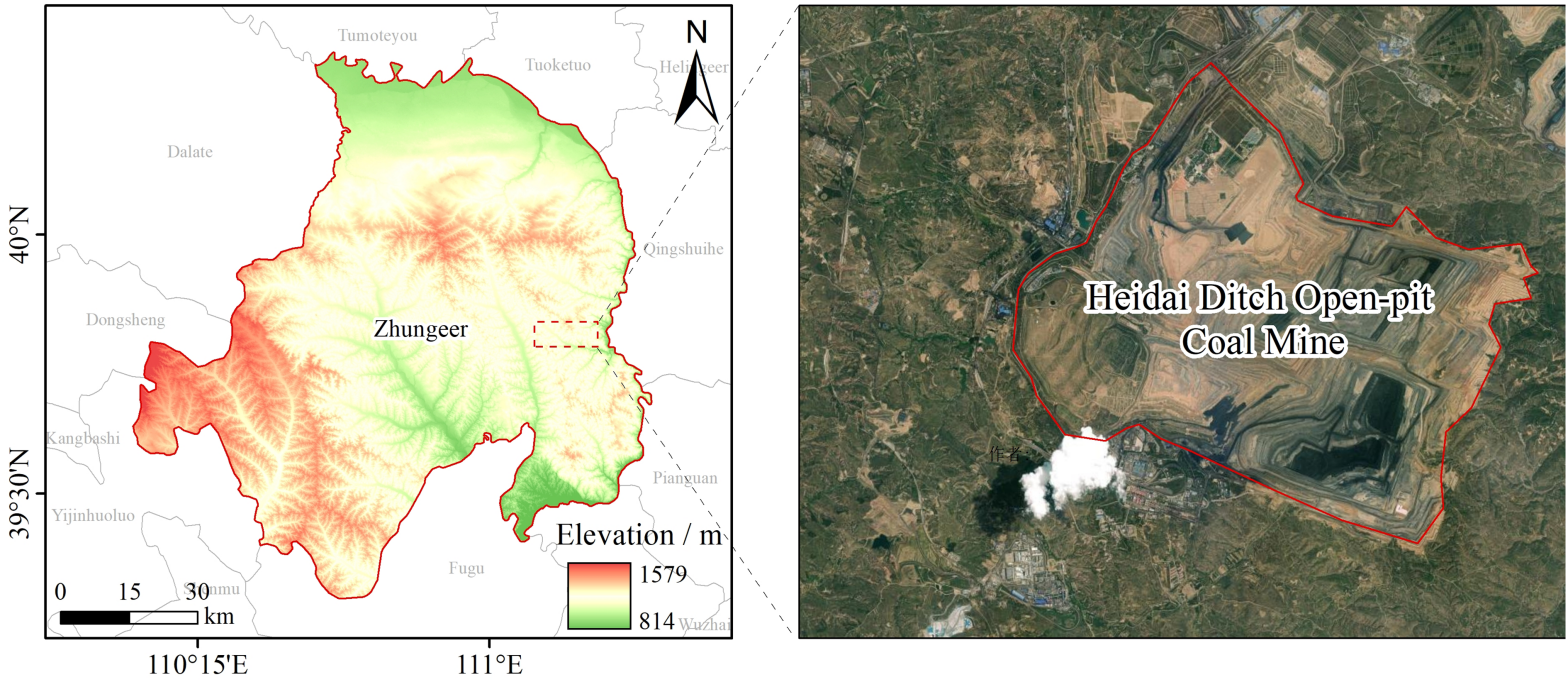
Map of study area. Geographical location and sampling distribution map of the study area is drawn by the research team based on the public geographic information data of the geospatial data cloud platform Map source credit: https://www.gscloud.cn.

The Haidaigou open-pit coal mine is located in the eastern part of Jungar Banner, Ordos City, Inner Mongolia Autonomous Region, China (39°43′–39°49′N, 111°13′–111°20′E), between Haidaigou and Longwanggou on the west bank of the Yellow River, and the Loess Plateau (Ordos Plateau) area at the border of Jin, Shan, and Meng, 130 km north of Hohhot (Fig. 1). With an elevation of 1,025–1,302 m, and a total area of 6,546 hectares, of which 259 hectares are industrial plaza, 197 hectares, 210 hectares, 170 hectares and 150 hectares are north, east, west and silver bay dumps respectively, and 5,560 hectares are inner dumps, reclaimed since 2008, 186.9 hectares by the end of 2011, and 968.2 hectares by the end of 2024, which are distributed in the terrace slopes at an altitude of 1,255 and 1,270 m. The landform of this area consists of loess hills and gullies, covering Paleozoic and Mesozoic strata, Tertiary clay, Quaternary loess, *etc*. The slightly alkaline, low-fertility loamy soil is widely distributed, and the soil of the landfill terrace has been compacted and backfilled by dump trucks. The average annual temperature is 7.2 °C, the average annual precipitation is 404.1 mm (60–70% in July–September, with heavy rainfall), the average annual wind speed is 3.6 m/s, and there are 42.2 days of windy days and 17–26 days with sandy days conditions each year. The vegetation is characteristic of the warm-temperate grassland zone, exhibiting sparse and short growth, with a coverage rate typically below 30%. This degraded ecological state, coupled with significant mining disturbances, highlights the pressing necessity for effective vegetation restoration strategies in the region.

### Sample preparation

The experiment was conducted in mid-August 2024 at the Heidaigou Open-pit Mine, where the inner dump platform was established at an elevation of 1,251 m. Under the same site conditions, Select five species three-year-old plants, namely *Caragana korshinskii*, *Hippophae rhamnoides*, *Hedysarum fruticosum*, *Medicago sativa* and *Astragalus adsurgens*. The dimensions of the sample plots were determined based on plant life forms and planting densities. The dimensions of the shrub plots for *Caragana korshinskii* and *Hippophae rhamnoides* were 50 m × 50 m, with three replicates for each. The afforestation methods employed for both *Hippophae rhamnoides* and *Caragana korshinskii* involved the use of seedlings, arranged with a row spacing of 1.5 m × 2 m. In contrast, the semi-shrub *Hedysarum fruticosum* and the two herbaceous plants were established through direct seeding, achieving a coverage of 60%. Each sample plot measured 10 m × 10 m and included three replicates. Twenty plants were randomly selected from each plant plot for measurement of plant height, crown width, and ground diameter, with subsequent calculation of their average values. To ensure representativeness and minimize individual variability, standard plants were systematically selected based on morphological proximity to the population mean. For each species, the average values of plant height, crown width (east–west and north–south), and ground diameter were calculated from all measured individuals across three replicate plots. Subsequently, Distance in this multi-trait space was computed for each plant relative to the species mean. The three plants with the smallest Distances in each replicate plot (*i.e*., those morphologically closest to the mean) were identified as standard plants. This approach yielded a total of nine standard plants per species (three per plot × three replicates), ensuring that the sampled roots reflected the typical growth characteristics of the population. All subsequent root excavation and mechanical tests were conducted on these designated standard plants. The growth status of the five plants and the selection of standard strains are presented in Table 1.

**Table 1:** Growth performance of five plant species and selection of standard plants.

Plant species	Plant height/cm	Crown/cm		Diameter/mm
		East-West	North-South	
*Caragana korshinskii*	118.23 ± 12.16	72.05 ± 9.18	74.12 ± 10.59	3.98 ± 1.29
*Hippophae rhamnoides*	87.73 ± 17.12	63.23 ± 17.36	66.56 ± 15.32	7.09 ± 2.16
*Hedysarum fruticosum*	120.02 ± 20.32	85.18 ± 27.61	82.90 ± 30.81	1.79 ± 0.12
*Astragalus adsurgens*	49.72 ± 10.28	60.12 ± 11.74	58.36 ± 10.38	2.92 ± 0.13
*Medicago sativa*	46.27 ± 9.68	52.37 ± 12.31	48.47 ± 12.34	3.03 ± 0.56

### Root excavation, collection, and analysis

Root collection focused on the underground root systems of the standard plants. Excavation was conducted on one side of each standard plant to a depth of 1.5–2.0 m. The contraction excavation method was Shan & Tao (1992) employed: soil was progressively cleared from the outer boundary 1 m from the plant base towards the center to minimize damage to the root system during excavation.

The root diameter grading standards established in this study are as follows (Bao, 2000): the roots of *Caragana korshinskii*, *Hedysarum fruticosum*, *Medicago sativa*, and *Astragalus adsurgens* were divided into three classes: 0–0.5, 0.5–1, and 1–1.5 mm; the roots of *Hippophae rhamnoides* were divided into two classes: 0.5–1 and 1–1.5 mm. After collection, the roots were immediately placed in black plastic bags, wrapped with moist sand for moisture retention, and transported to the laboratory within 24 h for subsequent processing.

### Soil sampling and analysis

#### Soil sampling scheme

In an area with consistent site conditions on the internal dump platform, a loess area without vegetation restoration was selected for soil sampling. Two soil profiles were excavated to a depth of 100 cm, and samples were collected at 20 cm intervals (*i.e*., 0–20, 20–40, 40–60, 60–80, and 80–100 cm layers). For each soil layer, three undisturbed soil samples were collected using the “needle” ring knife method (ring knife volume: 50 cm^3^), resulting in 15 undisturbed soil samples per profile and a total of 30 samples from two profiles. All undisturbed soil samples were sealed in aluminum boxes to prevent moisture loss; meanwhile, 5–7 kg of disturbed soil was collected from each layer and brought back to the laboratory for geotechnical testing.

#### Analysis of basic soil physicochemical properties

Particle Size Distribution: After air-drying, the undisturbed soil samples were passed through a 2 mm sieve, and the particle size distribution was determined using a Mastersizer 3000 laser particle size analyzer. According to the Kachinsky (Huang, 2000) soil texture classification system, the soil in this area was classified as light loam (Table 2). Sand (2~0.075 mm) accounts for about 35%, and clay (<0.075 mm) accounts for 54.23%. The whole has the characteristics of uniform texture, loose and porous, and high clay content, which is in line with the soil background characteristics of the study area.

**Table 2:** Table of results of analyses of mechanical composition of experimental soils.

Soil type	Soil mechanical composition%					
	>2 mm	2~1 mm	1~0.5 mm	0.5~0.25 mm	0.25~0.075 mm	<0.075 mm
Yellow soil	0.57	1.51	8.17	3.98	31.54	54.23

**Note:**

The loessial soil is a commonly used soil type for the reclamation of coal mine dumps in the region. It belongs to a kind of loess soil. Its formation is closely related to regional geological evolution, climatic conditions and soil formation. The parent material is derived from the Quaternary loess sediments and is rich in calcium carbonate. Under the influence of the warm temperate semi-arid climate (annual precipitation is about 400 mm, concentrated in July and September and heavy rains), it undergoes long-term wind transport deposition and mild chemical weathering. In addition, the low vegetation coverage (usually <30%) leads to weak soil formation and incomplete soil profile development, and finally forms the current characteristics.

Water Content determined using the 105 °C oven-drying method (drying duration: 24 h). The calculation formula is as follows:

(1) $${\omega }=\displaystyle{{{{m}_{0}}-{{m}_{d}}} \over {{{m}_{d}}}}\times {100 \rm \% }$$

where: ${\omega }$ is the natural soil water content (%); ${{m}_{0}}$ is the mass of the wet soil sample (g); and ${{ m}_{ d}}$ is the mass of the soil sample after drying to constant weight at 105 °C (g). The measured natural soil water content was 7.5%.

Bulk Density determined using the ring knife method. Combined with the water content data, the dry density was calculated using the following formula:

(2) $${\rho d }=\displaystyle{{\rho } \over {{1 + 0.01}{\omega }}}$$

where: ${\rho d}$ is the soil dry density (g/cm^3^); ${\rho }$ is the soil bulk density (g/cm^3^); ${\omega }$ is the natural soil water content (%); and 0.01 is the percentage conversion factor. The measured soil bulk density was 1.56 g/cm^3^, and the calculated dry density was 1.46 g/cm^3^.

Chemical Properties: Soil organic matter content: 3.77 g/kg; total N content was 0.862 g/kg, total P content was 0.324 g/kg, total K content was 8.89 g/kg, speed N content was 3.35 mg/kg, speed P content was 5.67 mg/kg, speed K content was 93.83 mg/kg.

Soil liquid limit and plastic limit tests were conducted using a liquid-plastic limit combined tester. The test data were interpreted according to the “Standard for Engineering Classification of Soil” (GB/T 50145-2007, Ministry of Water Resources of the People’s Republic of China, 2008). The loess used in this study contained over 50% fine particles by total soil mass, classifying it as fine-grained soil; furthermore, the coarse particle content within the fine-grained soil accounted for 25–50% of the total soil mass. The liquid limit and plastic limit are key physical indicators for cohesive soils and can be used to calculate the soil plasticity index (Ip) and liquidity index. The plasticity index is calculated as follows:

(3) $${{I}_{P}}={{W}_{L}}-{{W}_{P}}$$

where: ${{I}_{P}}$ is the plasticity index; ${{W}_{L}}$ is the liquid limit (%); and ${{W}_{P}}$ is the plastic limit (%).

The test results showed a soil plasticity index ${{I}_{P}}$ = 8.62 ( ${{I}_{P}}$ < 10) and a liquid limit ${{W}_{L}}$ = 27.04% ( ${{W}_{L}}$ < 50%). Based on the position of the plasticity index and liquid limit in the plasticity chart, this fine-grained soil was classified as silty sand with low liquid limit (MLS).

#### Sample preparation of root-soil composite

Root-soil composite samples were prepared following the methods of Zhang (2019) and Zhu (2016), controlling parameters such as natural soil water content, saturated water content, and dry density. Five soil water content gradients were established to simulate the natural soil moisture fluctuations in the study area. The set water content gradients were 3.5%, 7.5%, 11.5%, 15.5%, and 19.5%. Among these, 3.5% and 7.5% were the field-measured natural water contents. All gradients were below the soil saturated water content (21.8%), meeting the experimental requirements. The amount of water required to prepare samples at different water content gradients was calculated using the following formula:

(4) $${{m}_{\omega }}={{ m}_{ a}}\displaystyle{{{{ \omega }_{{tar}}}-{{\omega }_{a}}} \over {{1 + 0.01}{{\omega }_{a}}}}$$

where: mw is the required amount of water (g); ${{m}_{a}}$ is the mass of the air-dried soil sample (g); ${{\omega }_{a}}$ is the water content of the air-dried soil (%); ${{\omega }_{{tar}}}$ is the target water content (%); and 0.01 is the percentage conversion factor.

Roots with a diameter of 1–1.5 mm were embedded in the root-soil composite samples, and the soil dry density was controlled at 1.56 g/cm^3^, consistent with the field-measured value.

### Shear test of root-soil composite

A ZJ-type four-unit strain-controlled electric direct shear apparatus (Model: ZJ-2A, manufactured by Nanjing Soil Instrument Factory, China; shear force range: 0–50 kN; shear rate accuracy: ±0.01 mm/min) was used to conduct shear strength tests on root-soil composites. The test samples were root-soil composites constructed with roots of 1–1.5 mm diameter, with a natural soil water content of 7.5% and a dry density of 1.56 g/cm^3^. Based on field survey data of the density of the five plant root systems within the concentrated distribution layer per unit volume of soil, the number of roots in each root-soil composite sample was standardized to four. The vertical load gradients were determined based on the derivation of the soil self-weight stress formula, as follows:

(5) $${{\sigma }_{z}}=\mathop \sum \limits_{{ i = 1}}^{n} {{ \gamma }_{i}}{{ h}_{i}}$$

where: ${{\sigma }_{z}}$ is the soil self-weight stress at depth *z* (kPa); ${{\gamma }_{i}}$ is the natural unit weight of the i-th soil layer (kN/m^3^); ${{h}_{i}}$ is the thickness of the i-th soil layer (m); and ${n}$ is the number of soil layers within the calculated depth range.

Considering the characteristics of the root concentrated distribution layer (0–1.5 m), four vertical load gradients were finally determined: 12.5, 25, 50, and 100 kPa, with three replicates for each load gradient. The shear test employed a constant shear rate of 0.8 mm/min to simulate saturated soil conditions during rainfall and to analyze the soil pore water pressure response characteristics. Following the “Standard for Soil Test Methods” (Ministry of Water Resources of the People’s Republic of China, 1999), the average shear strength from the three replicate tests was taken as the shear stress value under the corresponding vertical load. The cohesion (*C*) and internal friction angle ( ${\varphi }$) of the root-soil composites were derived based on the Mohr-Coulomb strength criterion, using the following formula:

(6) $${{\tau }_{f}}={\sigma }\cdot { tan\varphi + C}$$

where: ${{\tau }_{f}}$ is the soil shear strength (kPa); ${\sigma }$ is the normal stress generated by the vertical load (kPa); ${\varphi }$ is the internal friction angle (°); and ${C}$ is the cohesion (kPa).

It should be noted that when shear deformation occurs in the root-soil composite under shear stress, frictional resistance exists not only between soil particles but also at the interface between soil particles and roots. Therefore, the measured internal friction angle ( ${\varphi }$) represents the comprehensive internal friction angle of the root-soil composite system.

### Data analysis

All experimental data were organized and calculated using Microsoft Excel 2021. Statistical analysis was performed using SPSS 26.0 software, and data visualization was completed using Origin 2022 software. The significance level for all statistical tests was set a α = 0.05.

#### Descriptive statistical analysis

Descriptive statistical analysis was conducted on key mechanical indicators (shear strength, cohesion, internal friction angle) of the five plant root-soil composites under five soil water content gradients and four vertical load conditions. The mean value (Mean) and standard deviation (SD) of each group of data were calculated to characterize the central tendency and dispersion of the data, providing foundational data support for subsequent statistical tests.

#### Analysis of variance (ANOVA)

One-way analysis of variance (ANOVA) was used to investigate the effects of different soil water content gradients on the shear strength, cohesion, and internal friction angle of the same plant root-soil composite under the same vertical load. Simultaneously, differences in mechanical indicators between different plant root-soil composites and bare soil under the same water content condition were analyzed. Before conducting ANOVA, the Shapiro-Wilk test was used to verify data normality, and Levene’s test was used to verify homogeneity of variances. If the data did not meet the assumptions of normality or homogeneity of variances, the non-parametric Kruskal-Wallis H test was used as an alternative analysis.

#### Multiple comparison tests

When the results of ANOVA or the Kruskal-Wallis H test indicated significant overall differences (*p* < 0.05), the Least Significant Difference (LSD) method was used for *post hoc* multiple comparisons to clarify pairwise differences between groups. When intergroup differences were significant (*p* < 0.05), different lowercase letters were used for labeling, providing a statistical basis for judging intergroup differences in the results analysis.

#### Correlation and regression analysis

Pearson correlation analysis was used to quantify the linear correlation between soil water content and the shear strength, cohesion, and internal friction angle of root-soil composites. Curve regression analysis was performed to fit the response curves of shear strength of different plant root-soil composites to changes in soil water content. The coefficient of determination (R^2^) was calculated to evaluate the goodness of fit of the regression models, with R^2^ > 0.90 indicating a good fit.

#### Principal component analysis (PCA)

Principal component analysis (PCA) was performed on a dataset containing soil water content and three mechanical indicators of the five plant root-soil composites. Principal components with eigenvalues greater than 1 (PC1, PC2) were extracted. By analyzing the contribution rates of each principal component and the loading matrix of the original variables, the correlation between soil water content and mechanical indicators, as well as the differences in mechanical characteristics among different plant root-soil composites, were clarified.

## Results

### Shear behavior of root-soil composite and its variation with vertical load

**Figure 2:**
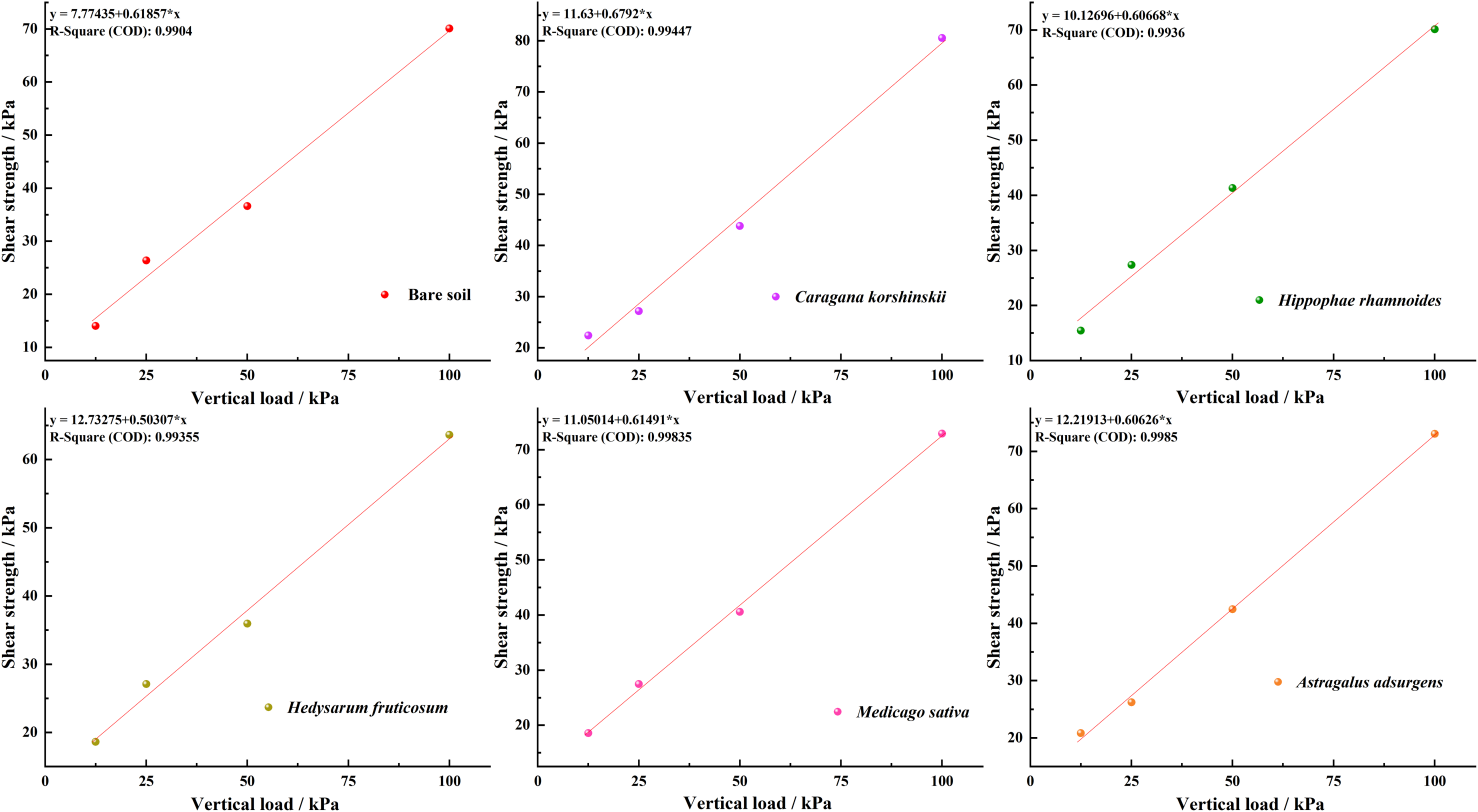
Relationship between shear strength of root-soil complexes and bare soils of five plant species and vertical loading.

As shown in Fig. 2, the relationship between shear strength and vertical load for five plant root-soil composites and bare soil without roots. The figure indicates that the shear strength of all five plant root-soil composites and bare soil increases with increasing vertical load, exhibiting a linear positive correlation. The multiple correlation coefficients were: bare soil, R^2^ = 0.990; *Caragana korshinskii* root-soil complex R^2^ = 0.995, *Hippophae rhamnoides* root-soil complex R^2^ = 0.994, *Hedysarum fruticosum* root-soil complex R^2^ = 0.994, *Medicago sativa* root-soil complex R^2^ = 0.998, *Astragalus adsurgens* root-soil complex R^2^ = 0.999, indicating that the linear regression fitting shear strength effect is good, in line with the Coulomb strength formula ${{\tau }_{ f}}={\sigma }\cdot { tan\varphi + C}$. It is proven that the shear relationship between bare soil and root-soil composite obeys the Mohr-Coulomb strength failure criterion of soil mechanics. The cohesion and internal friction angle of root-soil composite and bare soil can be calculated by this criterion, which is consistent with the research results of Cui et al. (2021). It can also be seen from the diagram that the shear strength of the root-soil complex of the five plants is compared with the bare soil, and the order of shear strength is as follows: *Hedysarum fruticosum* root-soil complex > *Astragalus adsurgens* root-soil complex > *Medicago sativa* root-soil complex > *Caragana korshinskii* root-soil complex > *Hippophae rhamnoides* root-soil complex > bare soil; the order of cohesion is *Astragalus adsurgens* 12.25 kPa ± 0.39b > *Hedysarum fruticosum* 11.59 kPa ± 0.43b > *Caragana korshinskii* 11.49 kPa ± 0.57a > *Medicago sativa* 11.06 kPa ± 0.41a > *Hippophae rhamnoides* 10.65 kPa ± 0.47b > soil cohesion 8.34 kPa ± 0.47a. The root-soil composite internal friction angle of *Caragana korshinskii*, *Hippophae rhamnoides*, *Hedysarum fruticosum*, *Medicago sativa*, and *Astragalus adsurgens* were 38.94° ± 3.22a, 33.21° ± 3.54b, 30.00° ± 2.85bc, 35.25° ± 3.75bd, 34.76° ± 3.39bd, respectively, and the internal friction angle of the soil was 40.11° ± 1.85ae; that is, the internal friction angle of the soil was not significantly different from that of the *Caragana korshinskii* root-soil complex (*p* < 0.05). The value of the internal friction angle of the other four plant root-soil complexes is smaller than the internal friction angle of the root-soil complex. Therefore, according to Cullen’s law, it can be assumed that cohesion plays an important role in the increase of shear strength of the root-soil composite. Therefore, according to Coulomb’s law, it can be concluded that cohesion plays an important role in improving the shear strength of the root-soil composite, which is consistent with the research results of Ming, Yao & Gao (2025).

**Figure 3:**
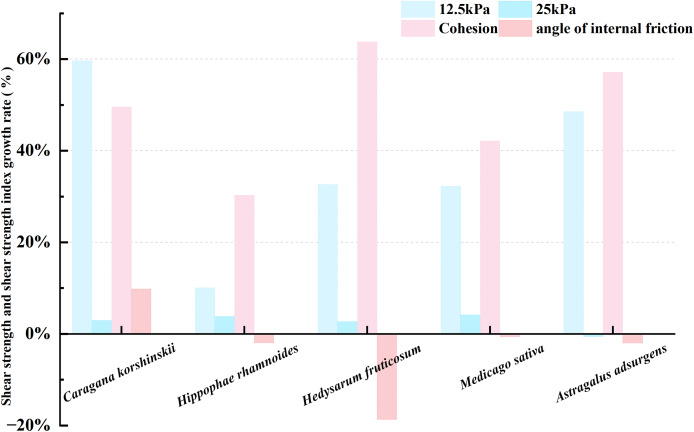
Plant root-soil composite shear strength and strength index increment.

As shown in Fig. 3. The 12.5 kPa load corresponds to about 80 cm of shallow soil with concentrated root distribution. The increment of the shear strength of the five plant root-soil complexes relative to the bare soil is in the order of *Caragana korshinskii* 60% > *Astragalus adsurgens* 50% > *Medicago sativa* 35% > *Hippophae rhamnoides* 33% > *Hedysarum fruticosum* 30%. This law reflects differences in the effects of different plant roots on soil shear strength improvement during their interaction with soil, and the analysis of the reasons is affected by plant root morphology, mechanical properties, and different bonding mechanisms with soil. The order of the increment of cohesion relative to bare soil is 46% of *Astragalus adsurgens*, >40% of *Caragana korshinskii*, >38% of *Hedysarum fruticosum* >32% of *Medicago sativa* >28% of *Hippophae rhamnoides*. Cohesion is a key index that reflects the bonding between soil particles. Plant roots enhance particle bonding by secreting organic cementing substances and by interspersing and winding soil particles, thereby increasing cohesion. The internal friction angle of the five plant root-soil composites showed a negative increment relative to the bare soil, which was *Caragana korshinskii*-3%, *Hippophae rhamnoides*-17%, *Hedysarum fruticosum*-25%, *Medicago sativa*-1% and *Astragalus adsurgens*-5%, respectively. The internal friction angle was related to surface roughness, particle gradation, and particle arrangement. After the plant roots were mixed into the soil, the original arrangement of the particles may be changed, or the root system itself may be relatively ‘flexible’. The characteristics of the root system reduce frictional occlusion between soil particles to some extent, making the internal friction angle appear negative. On the whole, cohesion plays a leading role in improving the shear strength of the root-soil composite, and different plants have different strengthening effects on soil shear strength. Under the load of 25 kPa corresponding to the soil pressure of about 150 cm, the increment of the shear strength of the five plant root-soil composites is ‘not significant’ compared with that of the bare soil. The reason is that in the soil layer of about 150 cm, the distribution of the five plant roots is ‘scarce or missing’, and the mechanical strengthening effect of the roots on the soil is difficult to play, and the effect of improving the shear strength of the soil is weakened.

### Effects of soil moisture content on the shear properties of root-soil composites of five plants

**Figure 4:**
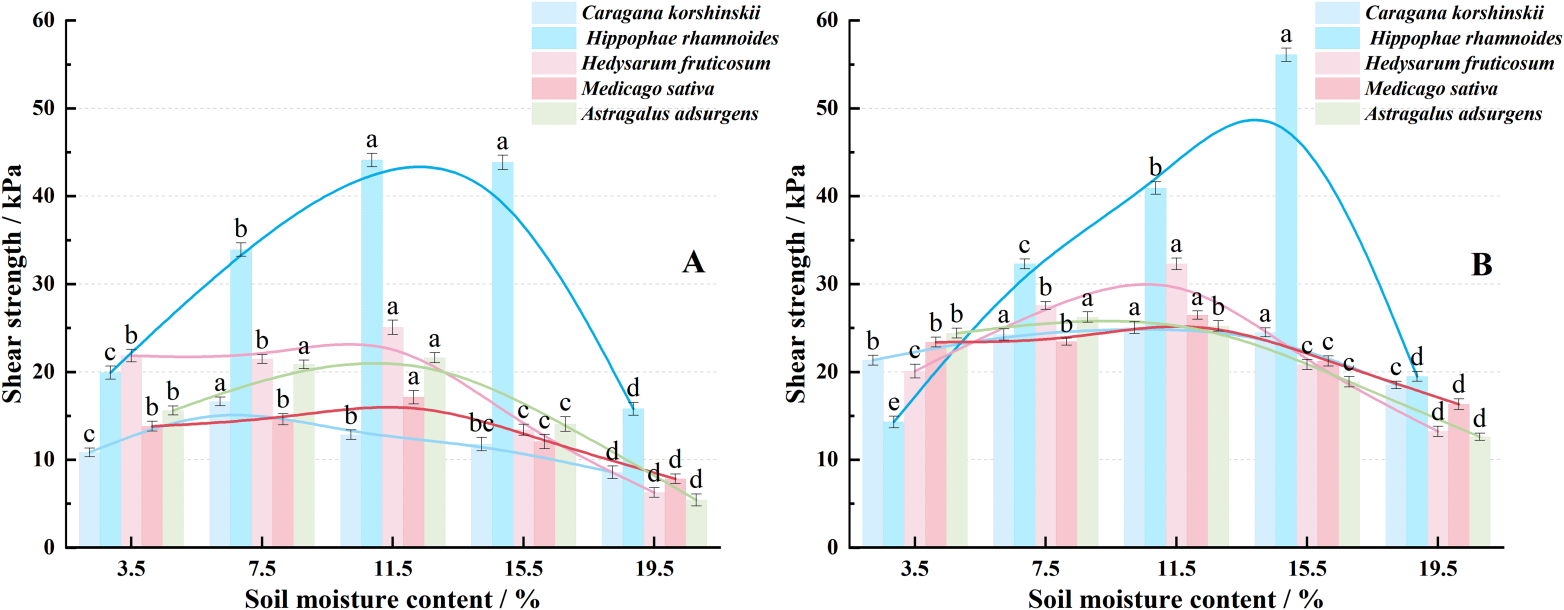
The variation trend of shear strength of five plant root-soil composites with soil moisture content under vertical load of 12.5 kPa (A) and 25 kPa (B).

Under a 12.5 kPa load, the peak shear strength of the *Caragana korshinskii* root-soil composite occurs at a soil moisture content of 7.5%. For *Hippophae rhamnoides*, *Hedysarum fruticosum*, *Astragalus adsurgens*, and *Medicago sativa* root-soil composites, the peak shear strength corresponds to a soil moisture content of 11.5%. When the load is increased to 25 kPa, the peak shear strength for *Caragana korshinskii*, *Hedysarum fruticosum*, and *Medicago sativa* root-soil composites is observed at a soil moisture content of 11.5%. In contrast, *Hippophae rhamnoides* root-soil composite reaches its peak shear strength at a soil moisture content of 15.5%, and *Astragalus adsurgens* at 7.5%. Variance analysis at the α = 0.05 level was used to compare the shear strength among root-soil complexes under different water contents and between root-soil complexes and bare soil. Results, shown with uppercase letters in Fig. 4A, indicate that the same letters denote no significant difference, while different letters indicate significant differences. The analysis demonstrates that the roots of the same plant species increase the soil’s shear strength. Additionally, soil shear strength changes significantly with varying soil moisture content. These findings show that, within a certain range, increasing soil moisture improves soil shear strength and mechanical stability. However, when soil moisture exceeds optimal values, plant transpiration helps regulate and reduce soil moisture, enhancing slope stability and decreasing the risk of geological disasters such as landslides. This highlights the superiority of biological forest and grass methods for soil erosion control from a biomechanical perspective.

The trend of shear strength of five plant root-soil composites with the increase of soil moisture content was fitted by curve regression. The fitting equation and determination coefficient are shown in Table 3, and the determination coefficient R^2^ > 0.90.

**Table 3:** Five regression equations of shear strength of root-soil complexes and bare soils of 35 plant species with changes in soil water content.

Shear strength	Regression equation	Coefficient of determination R^2^
12.5 kPa	y_*Caragana korshinskii*_ = −0.6967x^4^ + 8.9633x^3^ − 41.053x^2^ + 76.527x − 32.75	R^2^ = 1
	y_*Hippophae rhamnoides*_ = −6.8645x^2^ + 41.483x − 17.593	R^2^ = 0.9224
	y_*Hedysarum fruticosum*_ = −2.0524x^2^ + 8.349x + 15.111	R^2^ = 0.908
	y_*Medicago sativa*_ = −1.2379x^2^ + 5.9915x + 8.7133	R^2^ = 0.9054
	y_*Astragalus adsurgens*_ = −2.5993x^2^ + 12.851x + 5.5187	R^2^ = 0.9824
25 kPa	y_*Caragana korshinskii*_ = −1.365x^2^ + 7.653x + 14.81	R^2^ = 0.9483
	y_*Hippophae rhamnoides*_ = −3.5389x^3^ + 24.515x^2^ − 36.083x + 30.451	R^2^ = 0.9299
	y_*Hedysarum fruticosum*_ = −3.3319x^2^ + 17.927x + 5.6613	R^2^ = 0.9086
	y_*Medicago sativa*_ = −0.2178x^3^ + 0.6457x^2^ + 1.0935x + 21.563	R^2^ = 0.9037
	y_*Astragalus adsurgens*_ = −1.5314x^2^ + 6.1092x + 19.995	R^2^ = 0.9876

Under a vertical load of 12.5 kPa, the shear strength of the five plant root–soil complexes first increased, then decreased as soil moisture content rose (Fig. 4A). At 3.5% soil moisture, shear strength ranked as follows: *Hedysarum fruticosum* > *Hippophae rhamnoides* > *Astragalus adsurgens* > *Medicago sativa* > *Caragana korshinskii*. As the moisture content increased to 7.5% and 11.5%, the *Hippophae rhamnoides* composite showed the highest shear strength, reaching 33.91 and 44.11 kPa (*p* < 0.05), clearly above other plants. At these levels, tamarisk ranked second, indicating its reinforcing effect persists in moderately dry soil. Beyond 15.5% and 19.5% moisture, shear strength dropped sharply for all five complexes. High moisture content likely softened the soil and weakened interfacial cohesion.

Under a vertical load of 25 kPa (Fig. 4B), shear strength was generally higher than at 12.5 kPa. This indicates that external constraints enhance composite shear performance. Significant differences among plant species persisted (*p* < 0.05). At low moisture (3.5%), *Astragalus adsurgens*, *Medicago sativa*, and *Caragana korshinskii* exhibited higher shear strength. This reflects strong physical anchoring from dense fine roots in dry soil. At medium moisture (7.5–11.5%), *Hippophae rhamnoides* and *Hedysarum fruticosum* showed the strongest enhancements, reaching 40.93 and 32.30 kPa. When moisture exceeded 15.5%, shear strength declined for all composites. *Hippophae rhamnoides* maintained the highest value (56.09 kPa), but overall strength degradation was pronounced.

**Figure 5:**
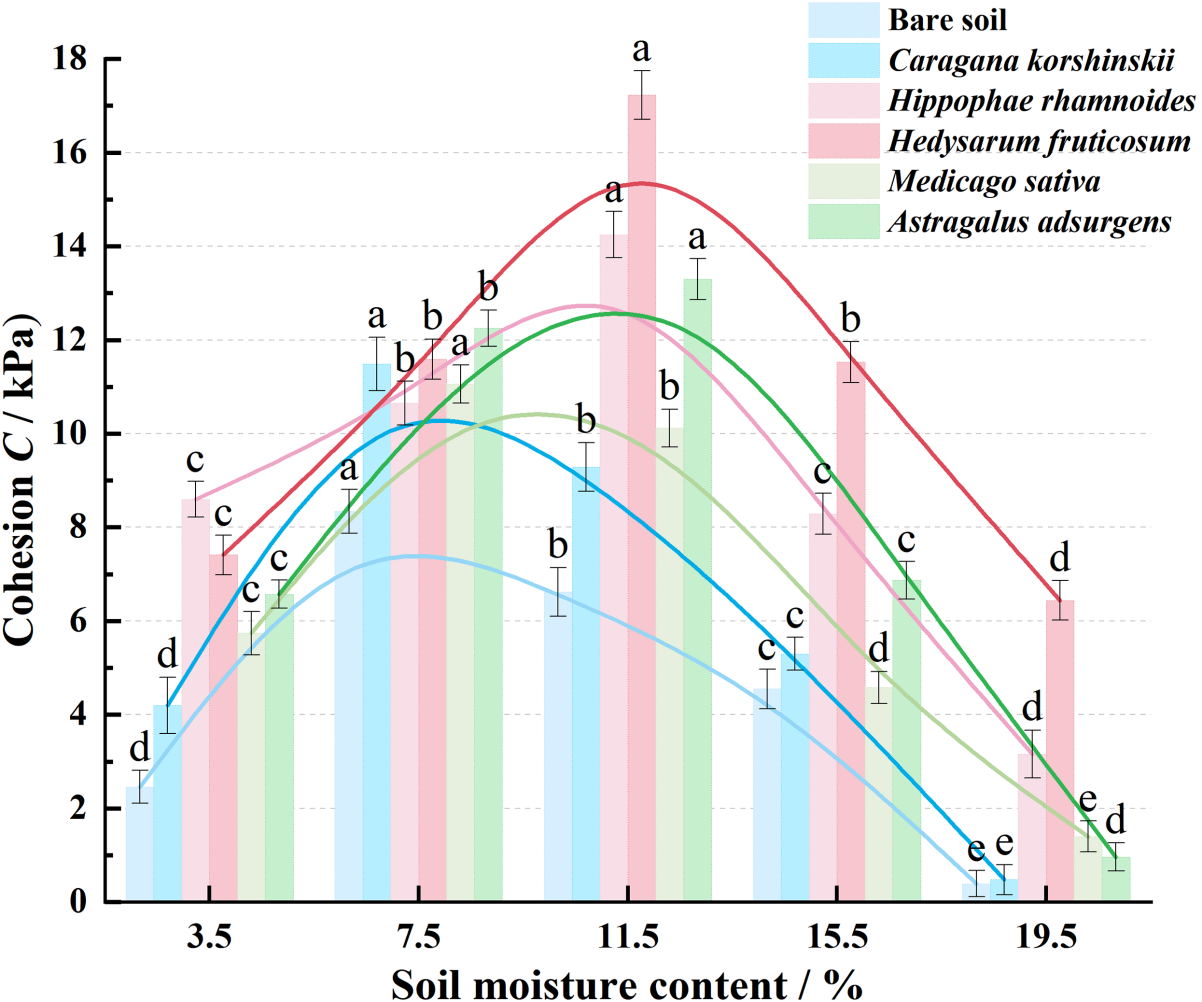
Trends of root-soil complexes and clay cohesion of bare soils of five plant species with soil water content.

**Figure 6:**
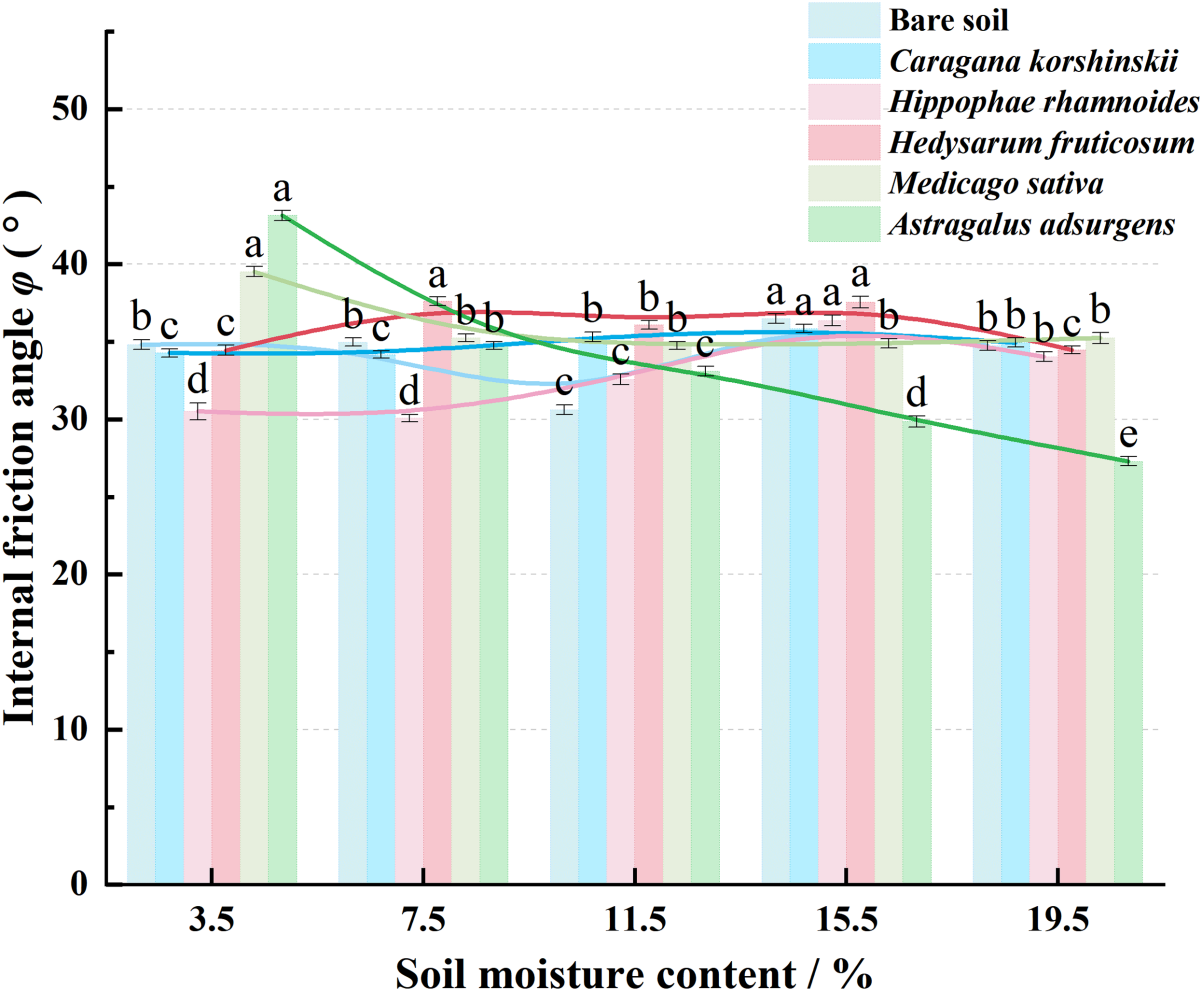
Trends of internal friction angle of root-soil complexes, bare soils of five plant species with soil water content.

As shown in Fig. 5, the cohesion of native soil and each plant root–soil composite increased first, then decreased with changing moisture. This mirrors the trend of shear strength. Peaks were primarily at 7.5% or 11.5% moisture, suggesting that moderate moisture levels enhance bonding between soil particles and the root–soil interface. Excessive moisture weakens this effect. At 3.5% moisture, *Hippophae rhamnoides* root-soil complex had the highest cohesion (8.60 kPa). This was followed by *Hedysarum fruticosum*, *Astragalus adsurgens*, *Medicago sativa*, and *Caragana korshinskii*, all higher than bare soil (2.46 kPa). At 7.5% moisture, *Caragana korshinskii* and its composite reached a peak (~12 kPa). At 11.5%, peak cohesion for these reached 14.25–17.23 kPa. Above 15.5% moisture, cohesion declined sharply but stayed generally higher than bare soil. Roots retain some binding and anti-sliding properties even at high moisture levels. Overall, roots boost soil cohesion significantly but have minimal impact on internal friction angle (Fig. 6). Regardless of moisture content, the internal friction angle changed little, indicating that roots primarily increase shear strength by increasing cohesion.

Curve regression fitting was conducted on the variation trends of the friction Angle and cohesion within five plant root-soil complexes with soil moisture content. The fitting equations and determination coefficients are shown in Table 4, with the determination coefficient R^2^ > 0.90.

**Table 4:** Five regression equations of root-soil complex cohesion and angle of internal friction of plants with soil water content change.

	Regression equation	Coefficient of determination R^2^
Cohesion C/kPa	y_bare soil_ = 0.4592x^3^ − 5.5918x^2^ + 18.799x − 11.078	R^2^ = 0.9713
	y_*Caragana korshinskii*_ = 0.7217x^3^ − 8.3529x^2^ + 26.815x − 14.888	R^2^ = 0.9914
	y_*Hippophae rhamnoides*_ = −0.06x^3^ − 1.1686x^2^ + 7.5114x + 2.01	R^2^ = 0.8985
	y_*Hedysarum fruticosum*_ = −0.0708x^3^ − 1.4968x^2^ + 10.934x − 2.31	R^2^ = 0.8801
	y_*Medicago sativa*_ = 0.7183x^3^ − 8.0079x^2^ + 24.694x − 11.74	R^2^ = 0.9937
	y_*Astragalus adsurgens*_ = 0.4292x^3^ − 6.0525x^2^ + 21.608x − 9.57	R^2^ = 0.983
Angle of internal friction *φ*/(°)	y_bare soil_ = −0.2583x^3^ + 2.7836x^2^ − 8.7081x + 41.468	R^2^ = 0.2131
	y_*Caragana korshinskii*_ = −0.2242x^3^ + 1.8589x^2^ − 4.0369x + 36.668	R^2^ = 0.993
	y_*Hippophae rhamnoides*_ = −0.7558x^3^ + 6.6232x^2^ − 15.431x + 40.166	R^2^ = 0.9823
	y_*Hedysarum fruticosum*_ = 0.0117x^3^ − 0.7821x^2^ + 4.3362x + 31.112	R^2^ = 0.6593
	y_*Medicago sativa*_ = −0.2975x^3^ + 3.3832x^2^ − 12.149x + 48.554	R^2^ = 0.9933
	y_*Astragalus adsurgens*_ = 0.7179x^2^ − 7.9641x + 49.628	R^2^ = 0.9638

### Five principal component analysis of mechanical properties of plant root-soil composite

**Figure 7:**
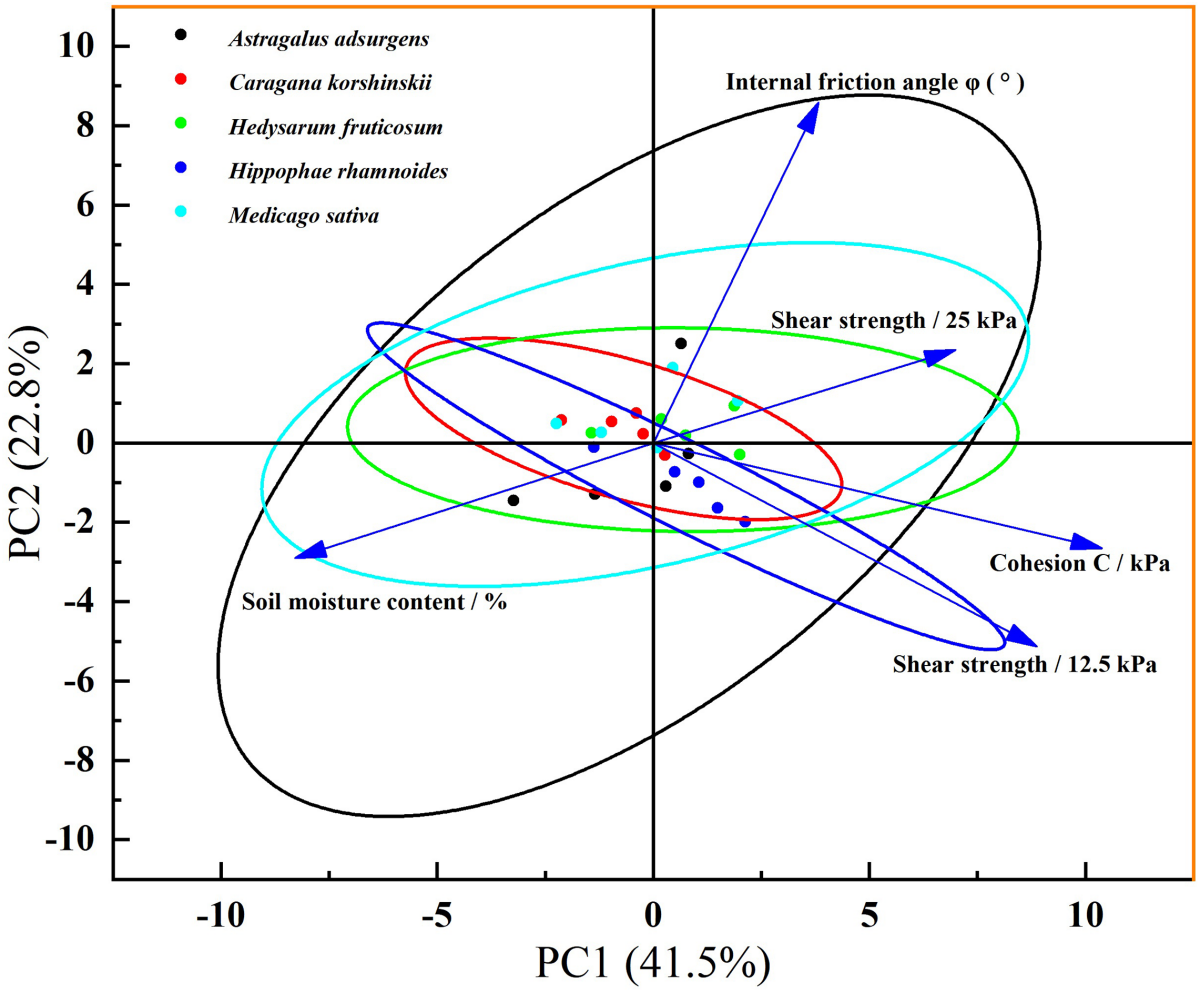
Five principal component analysis of mechanical properties of plant root-soil composite.

Figure 7 depicts the results of principal component analysis (PCA), where PC1 accounted for 41.5% and PC2 for 22.8% of the total variance. Together, these two axes explained 64.3% of the relationship between variables concerning the mechanical characteristics of root-soil composites, justifying the utilization of a two-dimensional ordination plot. PC1 primarily differentiates soil moisture content and mechanical properties: arrows representing soil moisture content point towards the negative PC1 direction, while those for internal friction angle, cohesion, and shear strengths (12.5/25 kPa) align with the positive PC1 direction. Thus, PC1 signifies a gradient where higher scores correspond to lower soil moisture and stronger root-soil mechanical properties, while lower scores indicate higher moisture and weaker properties. On the other hand, PC2 captures the residual variation not explained by PC1, showing minimal direct relevance to the moisture-mechanical property relationship. This distribution of arrows directly illustrates the influential role of soil moisture content in the mechanical interaction of root-soil systems. The plant species *Astragalus adsurgens*, *Caragana korshinskii*, *Hedysarum fruticosum*, *Hippophae rhamnoides*, *Medicago sativa*, and others clustered in the positive PC1 region exhibit a strong correlation with mechanical indices. This association suggests that these species demonstrate enhanced root-soil mechanical strength under low moisture conditions, indicating a superior adaptation to arid environments. In contrast, species located in the negative PC1 region display weaker moisture-mechanical connections, which may result from moderate responses to variations in soil moisture or a greater influence of factors such as root morphology. Therefore, soil moisture content emerges as the primary factor influencing the differences in shear strength, internal friction angle, and cohesion of root-soil composites. Distinct correlations exist between mechanical characteristics and environmental factors across different plant species, and future research could further elucidate the mechanisms underlying the interactions among plant traits, environmental factors, and mechanical properties.

## Discussion

### Interspecific differences and mechanisms of mechanical properties of root-soil complexes of different plants

The plant root-soil complex is an intricate and sophisticated structure composed of plant roots, soil particles, water, and air. These components interact synergistically to provide fundamental support for plant growth and soil stability. Under natural water content, there were significant differences in the increment of shear strength and cohesion of the five plant root-soil complexes compared with the increment of bare soil: *Caragana korshinskii* had the highest increment of shear strength (60%), *Astragalus adsurgens* had the highest increment of cohesion (46%), and *Hedysarum fruticosum* had the lowest increment of shear strength (29%) and cohesion (38%). This result was determined by root morphology and mechanical properties.

Due to the high degree of lignification and root length density, *Caragana korshinskii* (deep root shrub) restricts soil particles through the ‘interspersing-winding’ effect, and secretes organic cementing substances to enhance the root-soil interface bonding force (Gerile et al., 2018); the ‘network structure’ formed by 1~1.5 mm fine roots of *Astragalus adsurgens* (Leguminosae herb) disperses the shear stress and significantly improves the cohesion, which is consistent with the conclusion of ‘fine root network enhances cohesion’ by Fu, Tan & He (2021).

The root distribution of *Hedysarum fruticosum* (semi-shrub) is shallow, the diameter differentiation is not obvious, the tensile strength of single root is lower than that of *Caragana korshinskii*, the root density is lower than that of *Astragalus adsurgens*, and the soil fixation effect is weak, which is in line with the law of “root morphology determines soil fixation efficiency” by Cao et al. (2020). In addition, the internal friction angle of the root-soil composites of the five plants was lower than that of the bare soil (negative increment of −1% to −25%), and there was no significant correlation with the water content, indicating that the root system had a greater influence on the cohesion than the internal friction angle. Because the internal friction angle depends on the roughness and gradation of soil particles the “flexible” characteristics of 1~1.5 mm fine roots weaken the friction occlusion of particles (Xie, 2024), which further confirms that “in the root-soil composite dominated by fine roots, cohesion is the core factor to improve the shear strength” (Zhang, 2019; Gerile & Zuo, 2015).

### The regulation mechanism of soil water content on the mechanical properties of root-soil composites

The mechanical properties of soil-root complexes serve as core indicators for maintaining slope stability, ecological restoration effectiveness, and agricultural production safety. As one of the most dynamic soil state parameters, soil moisture content exerts significant regulatory effects on the mechanical properties of these complexes by altering root-soil interface interactions, soil structure, and root system characteristics. With the increase of soil moisture content, the shear strength and cohesion of the root-soil complexes of the five plants showed a trend of ‘first increase and then decrease’, and the peak values corresponded to different moisture contents: *Caragana korshinskii* and *Astragalus adsurgens* were 7.5% (*Astragalus adsurgens* can also be 11.5%), *Hippophae rhamnoides*, *Hedysarum fruticosum*, and *Medicago sativa* were 11.5%, reflecting the two-way regulation of water on root-soil interaction.

When the moisture content is low (3.5%), the shrinkage of soil particles increases the root-soil interface gap and decreases the cohesive force (Jiang et al., 2015). When the water content is suitable (7.5~11.5%), the particles expand and adhere to the roots, and the root cell wall softens to enhance the interfacial friction coefficient and bond strength, which is consistent with the phenomenon of Mei et al. (2023), ‘water promotes root-soil bonding’. When the water content exceeds the optimum value (15.5~19.5%), free water dilutes the organic cementing material, increases pore water pressure, and reduces the transfer efficiency of cohesive force and shear stress (Jiang et al., 2025). At this time, the high transpiration rate of shrubs such as *Hippophae rhamnoides* can maintain soil moisture stability, confirming the superiority of forest and grassland measures in preventing soil erosion (Zhang, 2025). The difference in the optimum water content of different plants (such as 7.5% of *Caragana korshinskii vs* 11.5% of *Hippophae rhamnoides*) is related to the water adaptability of their original habitats (Gerile et al., 2018; Lv et al., 2024), providing a basis for vegetation mixing.

### Biomechanical optimization potential of vegetation mixed configuration

Mixed vegetation configurations not only enhance biodiversity but also have the potential to synergistically improve system functionality beyond the sum of individual parts from a biomechanical standpoint. By employing scientifically curated plant combinations, there is a significant opportunity for optimizing ecological restoration, disaster management, and urban green spaces. This approach could serve as a pivotal strategy in advancing ecological security and fostering sustainable development. From a mechanical perspective, research indicates that a mixed forest root system may enhance the mechanical stability of surface soil more effectively than a monoculture forest. For instance, the combination of *Caragana korshinskii* (with shear strength advantage) and *Astragalus adsurgens* (with cohesion advantage) could compensate for the deficiencies in bare soil mechanics. Moreover, the varying optimal water content among different plant species (*Caragana korshinskii* 7.5%, *Hippophae rhamnoides* 11.5%, *Hedysarum fruticosum* 11.5%) suggests that mixed forests can sustain stability within a water fluctuation range of 3.5% to 19.5%, preventing abrupt declines in soil consolidation efficiency observed in single-species plantations. These findings align with the concept proposed by Sui’s (2021) regarding the enhancement of soil consolidation sustainability through mixed vegetation niche complementarity and offer initial mechanical validation for the shrub-herb mixed reclamation model proposed by Feng (2024) research.

Moreover, the spatial distribution of roots in mixed forests, exemplified by the deep roots of *Caragana korshinskii* and the shallow roots of *Astragalus adsurgens*, is posited to create a “stereo-soil-fixing network.” This hypothesis is consistent with Lu conclusion that the combination of deep-rooted shrubs and shallow-rooted herbs enhances the shear strength of various soil layers (Lu, 2023). Additionally, it is speculated that the mechanical reinforcement typically provided by individual plant roots under a 25 kPa load within a 150 cm soil layer may be compensated for by such a mixed root system. It is important to note that this study focused solely on roots within the 1 to 1.5 mm diameter class. Further investigation is required to assess the mechanical contributions of roots across different diameter classes (Fan & Su, 2008; Abdul Waheed, Xu & Murad Muhammad, 2025), as well as the impacts of root aging and the optimization of mixing ratios.

## Conclusions

(1) At natural moisture content, the maximum increase in shear strength of root-soil composites from five native plants relative to bare soil was 60% (*Caragana korshinskii*), while the minimum was 29% (*Hedysarum fruticosum*). The highest cohesion increase was 46% (*Astragalus adsurgens*), and the lowest was 28% (*Hippophae rhamnoides*). Across five moisture content gradients, cohesion in root-soil composites consistently exceeded that of bare soil, while internal friction angle showed no discernible pattern, indicating cohesion as the primary factor enhancing shear strength.
(2) With the increase of moisture content, the shear strength and cohesion of the five plant root-soil complexes showed a trend of “first increase and then decrease”, and there was a species-specific optimum moisture content: *Caragana korshinskii* was 7.5%, *Hippophae rhamnoides*, *Hedysarum fruticosum*, *Medicago sativa* was 11.5%, *Astragalus adsurgens* was 7.5% or 11.5%. The appropriate moisture content improves soil stability by enhancing the cohesive force at the root-soil interface; at a suitable water content, plant transpiration can adjust soil moisture and reduce landslide risk, reflecting the soil and water conservation advantages of forest and grass measures.
(3) The differences in mechanical properties of different plant root-soil composites (such as the shear advantage of *Caragana korshinskii* and the cohesion advantage of *Astragalus adsurgens*) and the complementarity of optimum water content make the mixed forest form a “three-dimensional soil consolidation network” and maintain high soil stability within a large range of water fluctuations. The results provide a biomechanical optimization strategy for the mixed configuration of “shrub-semi-shrub-herb” in desertification control and coal mine dump reclamation in central and western Inner Mongolia.

It should be noted that this study has limitations: the differences between artificial root distribution and natural root distribution, the interaction of soil physical and chemical factors, and the focus on short-term effects of young plants. Future research should focus on mechanical properties of multi-root systems, coupled numerical simulation and experimental verification, and the synergistic mechanisms of ecological stabilizers and roots to improve the evaluation and reinforcement of slope stability in practical engineering applications.

## Supplemental Information

10.7717/peerj.21211/supp-1 Supplemental Information 1 Raw data. OPJU files can be opened using Origin 2022 (https://www.originlab.com/).

## Additional Information and Declarations

### Data Availability

The following information was supplied regarding data availability:

The raw data is available in the Supplemental File.
